# *Wolbachia* Promotes an Anti-Angiogenic Response Using an In Vitro Model of Vascular Endothelial Cells in Relation to Heartworm Disease

**DOI:** 10.3390/pathogens13070603

**Published:** 2024-07-22

**Authors:** Manuel Collado-Cuadrado, Claudia Alarcón-Torrecillas, Iván Rodríguez-Escolar, Alfonso Balmori-de la Puente, Elena Infante González-Mohino, Miguel Pericacho, Rodrigo Morchón

**Affiliations:** 1 Zoonotic Diseases and One Health Group, Faculty of Pharmacy, University of Salamanca, 37007 Salamanca, Spain; manuelcollado@usal.es (M.C.-C.); ivanrodriguez@usal.es (I.R.-E.); alfonso.balmori@ibe.upf-csic.es (A.B.-d.l.P.); elena.igm4@usal.es (E.I.G.-M.); 2 Centre for Environmental Studies and Rural Dynamization (CEADIR), University of Salamanca, 37007 Salamanca, Spain; 3 Biomedical Research Institute of Salamanca (IBSAL), University of Salamanca, 37007 Salamanca, Spain; claudia3alarcon@usal.es (C.A.-T.); pericacho@usal.es (M.P.); 4 Department of Physiology and Pharmacology, University of Salamanca, 37007 Salamanca, Spain

**Keywords:** anti-angiogenesis, *Wolbachia* sp., rWSP, *Dirofilaria immitis*, endothelial cells

## Abstract

Heartworm disease caused by *Dirofilaria immitis* is a vector-borne zoonotic disease responsible for the infection of mainly domestic dogs and cats, or these are those for which the most data are known. Humans are an accidental host where a benign, asymptomatic pulmonary nodule may originate. *Dirofilaria immitis* also harbours the endosymbiont bacteria of the genus *Wolbachia*, which play a role in moulting, embryogenesis, inflammatory pathology, and immune response. When *Wolbachia* sp. is released into the bloodstream, endothelial and pulmonary damage is exacerbated, further encouraging thrombus formation and pulmonary hypertension, facilitating congestive heart failure and death of the animal. Previous studies have shown that parasite excretory/secretory products are able to activate the pro-angiogenic pathway (formation of new vessels) to facilitate parasite survival. The aim of this study was to analyse the role of *Wolbachia* sp. and its relationship with the cellular processes and the angiogenic pathway in a model of human endothelial cells in vitro. The use of recombinant *Wolbachia* Surface Protein (rWSP) showed that its stimulation exerted an anti-angiogenic effect by detecting an increase in the production of VEGFR-1/sFlt1 and sEndoglin and did not affect the production of VEGFR-2 and mEndoglin (pro-angiogenic molecules). Furthermore, it did not stimulate cell proliferation or migration, although it did negatively stimulate the formation of pseudocapillaries, slowing down this process. These cellular processes are directly related to the angiogenic pathway so, with these results, we can conclude that *Wolbachia* sp. is related to the stimulation of the anti-angiogenic pathway, not facilitating the survival of *D. immitis* in vascular endothelium.

## 1. Introduction

Cardiopulmonary dirofilariosis (heartworm disease) is a vector-borne zoonotic disease caused by the nematode parasite *Dirofilaria immitis*, which mainly affects domestic canids and felids and a wide variety of wild hosts such as wolves, foxes, ferrets, lynxes, among others, where humans can also be infected, being an accidental host [1,2,3]. It is a cosmopolitan disease and occurs mainly in areas with moderate temperature and high humidity, with semi-tropical and tropical climates. It is widely distributed in all continents, although in those countries where it has been studied in greater depth, it has been reported that its distribution is not homogeneous, as is the case in the EEUU and Europe [1,2].

This parasite harbours endosymbiont bacteria of the genus *Wolbachia*, Gram-negative bacteria within the alphaproteobacteria, in the same way as some filarioids of the subfamilies Onchocercinae [4]. *Wolbachia* sp. is found in all stages of *D. immitis*, mostly in the hypodermal cords and reproductive tract of females and participates in the moulting and embryogenesis of the parasite. It is a key component in the development of inflammatory pathology and in the activation of the Th_1_ immune response, stimulating clot production and having an antifibrinolytic effect, favouring TxB_2_ (vasoconstrictive) and LTB_4_ (chemotactic) production on the vascular endothelium and having an antifibrinolytic effect [2,3,4,5,6,7,8,9,10]. In addition, *Wolbachia* sp., through the use of the *Wolbachia* Major Surface Protein (WSP), is used in the diagnosis of human and feline dirofilariosis [1,2].

Heartworm disease is a chronic progressive disease, mainly vascular and pulmonary, resulting from the presence of *D. immitis* in the pulmonary artery and right ventricle of the heart [9]. In parallel, acute pathology results from the death of adult *D. immitis* worms, naturally or by adulticidal treatment, and the molecules of the parasite and *Wolbachia* sp. are released into the bloodstream, exacerbating changes in the vascular endothelium (proliferative endoarteritis, narrowing of the endothelial lumen, deformation and loss of elasticity, and precapillary pulmonary hypertension), increasing thrombus formation and accelerating possible congestive heart failure [10]. Vascular anatomical changes lead to luminal obstruction, decreased blood flow, hypoxia, oedema and pulmonary hypertension, among others [10].

Angiogenesis is a dynamic process involving the formation of new blood vessels from pre-existing ones, which can occur normally during embryogenesis and also in pathological situations in response to stimuli such as hypoxia, inflammation, or tissue injury [11]. VEGF-A is the main isoform and a key mediator in angiogenesis that signals through VEGFR-2 receptors, resulting in a pro-angiogenic response [12] or VEGFR-1. Its soluble form, sFlt1, produces a negative regulation by binding to VEGF and preventing its binding to the VEGR-2 receptor, thus decreasing pro-angiogenic signals [13]. Endoglin (vascular protein) plays a fundamental role in angiogenesis and vascular remodelling too. In particular, when high concentrations of soluble endoglin (sEndoglin) are detected, it is attributed as an anti-angiogenic element, as it occurs in patients with cancer or cardiac pathologies with proangiogenic properties when membrane endoglin (mEndoglin) is detected [14].

*Dirofilaria immitis* excretory/secretory antigen has been shown to trigger a pro-angiogenic response, in addition to cell proliferation, cell migration, and the formation of pseudocapillaries [15], similar to that produced by *Trichinella spiralis* in the formation of encapsulated larvae [16,17] and *D. repens* in the formation of subcutaneous nodules [18]. However, preliminary studies indicate that *Wolbachia* sp. promotes the anti-angiogenic pathway under hypoxia [19]. The aim of this study, in relation to cardiopulmonary dirofilariosis, was to investigate the angiogenic pathway triggered by *Wolbachia* sp., using an in vitro model of vascular endothelial cell and the cellular processes it may trigger, with regard to the expression of angiogenic factors, cell proliferation, and migration.

## 2. Methods

### 2.1. Reagents and Cell Culture of Endothelial Cells

Recombinant *Wolbachia* Mayor Surface Protein (rWSP) was prepared and purified according to the process described by Brattig et al. [20] and Diosdado et al. [21]. Protein concentration was measured by DC protein assay commercial kit (Bio-Rad) and was stored at −80 °C. WSP was tested for the presence of endotoxin contamination using a quantitative *Limulus* amebocyte lysate test (LAL test QCL 1000; <0.4 U/mg protein; BioWhittaker, Walkersville, MD, USA). The endotoxin quantity was under the sensitivity level of cell stimulation (<0.4 U/mg protein). This protein was used because, with regard to cardiopulmonary dirofilariosis, it is related to proinflammatory pathology and increased severity of the disease, stimulating clot production, favouring vasoconstrictor and chemotactic function of the vascular endothelium and having an antifibrinolytic effect [2,3,4,5,6,7,8,9,10].

Human Umbilical Vein Endothelial Cells (HUVECs) were maintained in cell culture according to the methodology described by Rossi et al. [22]. In short, HUVEC were grown on 0.1% gelatine (Sigma-Aldrich, St. Louis, MO, USA), 0.01% fibronectin (Sigma-Aldrich), and 0.01% collagen (Corning, Corning, NY, USA)-coated wells using EBM-2 medium supplemented with SingleQuots^®^ (Lonza, Basel, Switzerland) and 10% fetal bovine serum. HUVECs were cultured at 37 °C/5% CO_2_ and passaged every 3 days.

### 2.2. Stimulation of Endothelial Cells, Cell Viability, and Cytotoxicity Assays

HUVECs were treated following the methodology described by Machado et al. [15]. In short, endothelial cells (10^6^ cells/plate) were plated on 35 mm culture plates and were grown for 3 days to obtain confluent cultures and treated with 1 μg/mL of 3 different stimuli: rWSP, recombinant Vascular Endothelial Growth Factor protein (VEGF-A) (R&D Systems), and rWSP plus VEGF-A. Non-stimulated cells were used as controls in the same conditions. VEGF-A was added to simulate the initiation of the angiogenic process in the vascular endothelium, which may subsequently derive in an anti- or pro-angiogenic process, depending on the stimuli produced together with VEGF-A [15,18].

Cell viability was analysed through cell counts using a Countess^®^ Automated Cell Counter (Invitrogen, Waltham, MA, USA) following the manufacturer’s instructions. Cytotoxicity was assessed in the supernatant of the stimulated and control cell cultures by a Toxilight BioAssay Kit (Cambrex, Verviers, Belgium) following commercial instructions. This commercial kit quantitatively measures the release of adenylate kinase from damaged cells.

### 2.3. Pro- and Anti-Angiogenic Factors Assays

VEGF-A, soluble VEGFR-1 (VEGFR-1/sFlt1), VEGFR-2, and soluble Endoglin (sEndoglin) concentrations were measured in the supernatants, and membrane Endoglin (mEndoglin) concentration was measured in the cell listed in HUVEC-stimulated and unstimulated cultures for the first 24 h by ELISA using a Human VEGF-A, VEGFR-1/sFlt1, VEGFR-2, and Endoglin Quantikine ELISA kit (R&D Systems, Minneapolis, MN, USA) following the manufacturer’s instructions. The results were presented as the mean ± SD of three experiments performed in triplicate. The supernatants of HUVECs were directly collected, and cell lysates were obtained with an ice-cold lysis buffer (20 mM Tris-HCl, 140 mM NaCl, 10 mM EDTA, 10% glycerol, 1% Igepal CA-630, pH 7.5) with a proteinase inhibitor cocktail (aprotinin, pepstatin, and leupeptin at 1 μg/mL each; 1 mM phenylmethylsulfonyl fluoride and 1 mM sodium orthovanadate). Finally, protein concentrations were measured by a DC protein assay commercial kit (Bio-Rad, Hercules, CA, USA) and were stored at −80 °C.

### 2.4. Cell Proliferation and Migration and Pseudo-Capillary Formation

Proliferation, migration, and endothelial cell tube formation assays were assessed as previously described [15,18] with some modifications. In short, cell proliferation was analysed over 10 days every 2 days by an MTT-based assay; cell migration was measured at 6 h by wound healing technique using mitomycin (Sigma) to inhibit cell division and favour migration; and pseudo-capillary tube formation was analysed in an Ibidi Angiogenesis Slide of 15 wells (0.23 cm^2^) with Matrigel^®^ (Corning) for 4 h evaluating the cell junctions (connections) and the cellular set (converging strengths of different cellular ramifications) that emerged in stimulated and unstimulated cell cultures.

### 2.5. Statistical Analysis

The GraphPad Prism v.7 was used for all data analyses. Analyses were performed by ANOVA and corrected for repeated measurements when appropriate. If ANOVA revealed overall significant differences, individual means were evaluated post hoc using Tukey’s test. All results were expressed as the mean ± SEM. In all experiments, a significant difference was defined as a *p*-value of *<* 0.05 or 0.01 for a confidence level of 95 or 99%, respectively.

## 3. Results

### 3.1. Effect on Cell Viability and Cytotoxicity and Pro- and Anti-Angiogenic Factors

No differences were found in the cell viability and cytotoxicity of the stimulated cultures with WSP, VEGF-A, and WSP + VEGF-A compared to the non-stimulated cell cultures.

**Figure 1:**
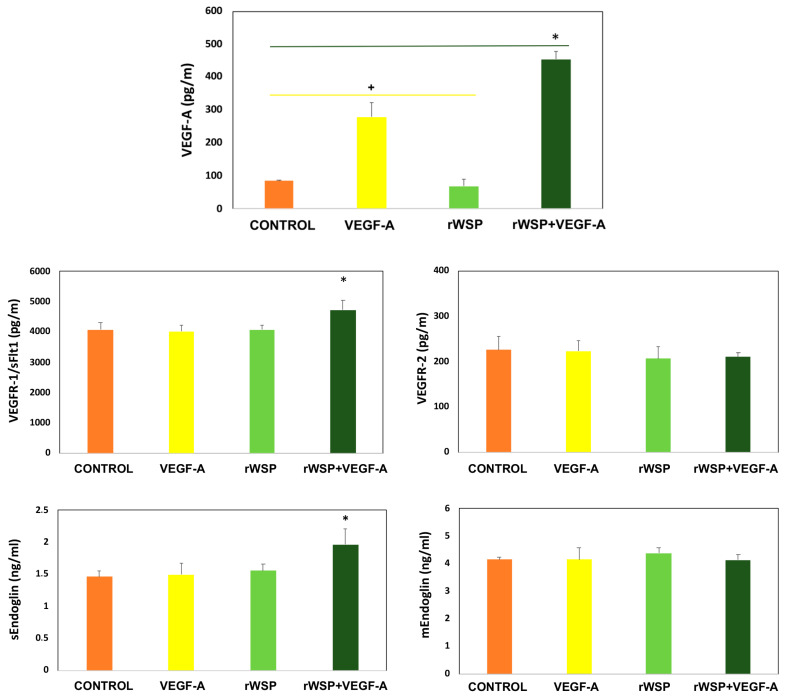
Effects of rWSP on VEGF-A, VEGFR-1/sFlt1, VEGFR-2, sEndoglin, and mEndoglin production in unstimulated cultures (●) and cultures stimulated with VEGF-A (●), rWSP (●), and rWSP + VEGF-A (●). Results are expressed as the mean ± SEM of 3 independent experiments. The asterisk (*) and the cross (+) indicate significant differences (*p* < 0.05) (rWSP: Recombinant *Wolbachia* Mayor Surface Protein; VEGF-A: Vascular Endothelial Grown Factor-A; VEGFR1/sFlt1; soluble Vascular Endothelial Grown Factor Receptor 1; VEGFR-2: soluble Vascular Endothelial Grown Factor Receptor 2; sEndoglin: soluble eEndoglin; mEndoglin: membrane Endoglin).

The cell cultures stimulated with rWSP + VEGF-A produced a significant increase in VEGF-A production compared to the cell cultures stimulated with rWSP (*t* = 5.914, *df* = 4, *p* = 0.0041) or VEGF-A (*t* = 5.914, *df* = 4, *p* < 0.0041), including the non-stimulated cultures (*t* = 25.00, *df* = 4, *p* < 0.0001), for the first 24 h (Figure 1). In addition, there were also significant differences between the cell cultures stimulated with VEGF-A, the cell cultures stimulated with rWSP (*t* = 7.518, *df* = 4, *p* = 0.0017), and the non-stimulated cultures (*t* = 7.718, *df* = 4, *p* = 0.0015). No significant differences between the cell cultures stimulated with rWSP and the non-stimulated cultures were detected, obtaining similar values.

Only the cell cultures stimulated with rWSP + VEGF-A also produced a significant increase in VEGFR-1/sFlt1 production compared to the cell cultures stimulated with rWSP (*t* = 3.206, *df* = 4, *p* = 0.0327) or VEGF-A (*t* = 3.265, *df* = 4, *p* = 0.0309), including the non-stimulated cultures (*t* = 2.835, *df* = 4, *p* = 0.0471). There were no significant differences between the cell cultures stimulated with rWSP or VEGF-A, or the non-stimulated cultures, for the first 24 h. In addition, in relation to VEGFR-2, no significant differences were observed between the cell cultures stimulated with the different reagents and the unstimulated cell cultures for the first 24 h (Figure 1).

Finally, the cell cultures stimulated with rWSP + VEGF-A also produced a significant increase in sEndoglin production compared to the cell cultures stimulated with rWSP (*t* = 7.652, *df* = 4, *p* = 0.0016) or VEGF-A (*t* = 5.634, *df* = 4, *p* = 0.0049), or the non-stimulated cultures (*t* = 11.04, *df* = 4, *p* = 0.0004), for the first 24 h. There were no significant differences between the cell cultures stimulated with rWSP or VEGF-A, or the non-stimulated cell cultures, for the first 24 h. In addition, no significant differences were observed between the cell cultures stimulated with the different reagents and the unstimulated cell cultures for the first 24 h in relation to mEndoglin (Figure 1).

### 3.2. Effect on Cell Proliferation and Migration and Pseudo-Capillary Formation

**Figure 2:**
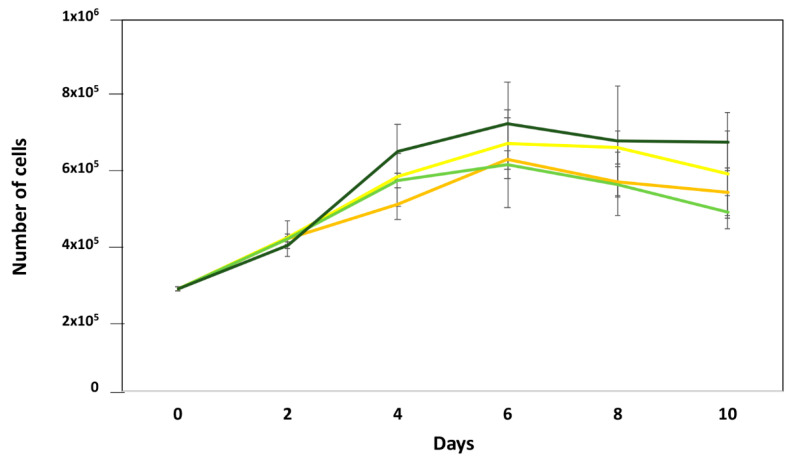
Effects of rWSP on cell proliferation in unstimulated cultures (●) and cultures stimulated with VEGF-A (●), rWSP (●), and rWSP + VEGF-A (●). Results are expressed as the mean ± SEM of 3 independent experiments (rWSP: Recombinant *Wolbachia* Mayor Surface Protein; VEGF-A: Vascular Endothelial Grown Factor-A).

**Figure 3:**
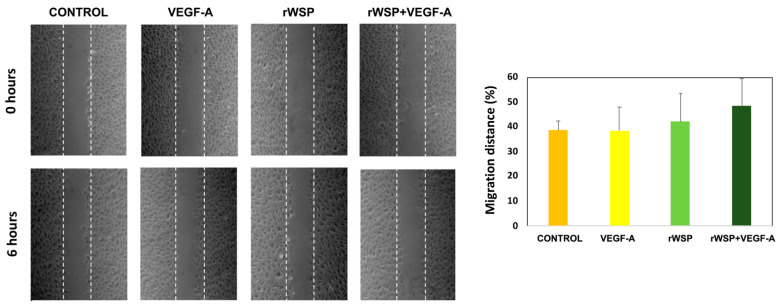
Effects of rWSP on cell migration distance in unstimulated cultures (●) and cultures stimulated with VEGF-A (●), rWSP (●), and rWSP + VEGF-A (●). Results are expressed as the mean ± SEM of 3 independent experiments (rWSP: Recombinant *Wolbachia* Mayor Surface Protein; VEGF-A: Vascular Endothelial Grown Factor-A).

**Figure 4:**
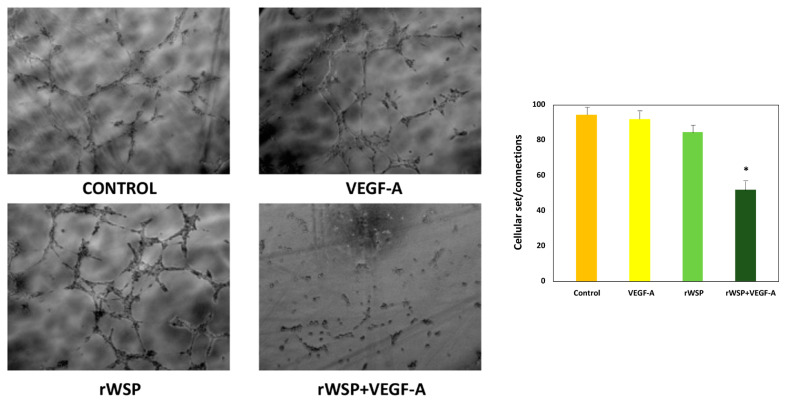
Effects of rWSP on connections and cellular set in unstimulated cultures (●) and cultures stimulated with VEGF-A (●), rWSP (●), and rWSP + VEGF-A (●). Results are expressed as the mean ± SEM of 3 independent experiments. The asterisk (*) indicates significant differences (*p* < 0.01) (rWSP: Recombinant *Wolbachia* Mayor Surface Protein; VEGF-A: Vascular Endothelial Grown Factor-A).

On cell proliferation, all cell cultures, stimulated and unstimulated, showed the typical curves of cell growth with progressive growth between days 0 and 6, experiencing a decrease in viable cells from day 8 until day 10. No significant differences were found between the cell cultures stimulated with rWSP + VEGF-A and rWSP or VEGF-A, including the non-stimulated cultures, in the number of viable cells (Figure 2). When cell migration was studied, there were also no differences in the treated and untreated cell cultures, although there was a slight increase in the stimulation with rWSP + VEGF-A (Figure 3). However, the formation of pseudo-capillaries and the connections/set relationship in the cell cultures stimulated with rWSP + VEGF-A showed a significant decrease compared to the cell cultures stimulated with rWSP (*t* = 12.40, *df* = 4, *p* = 0.0002), VEGF-A (*t* = 9.49, *df* = 4, *p* = 0.0007) and the non-stimulated cultures (*t* = 12.86, *df* = 4, *p* = 0.0002) (Figure 4).

## 4. Discussion

Angiogenesis is one of the most important processes related to the vascular system, particularly in response to stimuli such as hypoxia and/or vascular obstruction or rupture. It derives from the presence of thromboembolism or other obstructive or disruptive processes [23]. Furthermore, its stimulation leads to vasodilation, increased growth, and the remodelling of the vascular network and the differentiation of endothelial cells, which line the inner walls of blood vessels, in response to the presence of nitric oxide and increased permeability due to VEGF [24].

There are different studies where the angiogenic process is analysed in relation to different parasitosis caused by nematodes. On the one hand, encapsulated *T. spiralis* larvae in muscle tissue stimulate the attraction of blood microvessels, establishing a long-term relationship until ingestion by a new definitive host [16,17,25,26]. In addition, the excretory/secretory antiogens of the *D. immitis* adult worms activate the pro-angiogenic pathway and also the processes of cell migration and proliferation, as well as pseudo-capillary formation [15,27]. On the other hand, *Wolbachia* sp., endosymbiont bacteria of *D. immitis,* is able to stimulate in a hypoxic model of canine endothelial cells the anti-angiogenic pathway with the production of VEGF-A, but the cellular processes are not deepened [19]. Furthermore, in lymphatic parasitosis caused by *Brugia malayi*, *Onchocerca volvulus,* and *Wuchereria bancrofti*, their microfilariae and adult form promote lymphangiogenesis, in vitro remodelling of the lymphatic system, and modify the expression of angiogenic factors, thereby promoting vascular repair in damaged tissues, mainly when *Wolbachia* sp. is released upon natural death or by treatment of parasitic forms [28,29,30,31,32]. Our aim was to further investigate the effect of *Wolbachia* sp., using the rWSP, in an in vitro model of human endothelial cells in relation to the angiogenic pathway and the cellular processes that derive from it, such as cell migration and proliferation.

Our first results confirmed the existence of an anti-angiogenic effect, previously reported by Zueva et al. [19] in a hypoxic model of canine endothelial cells where they showed an increase in VEGF-A and sEndoglin (anti-angiogenic factor). In our model with human endothelial cells stimulated with VEGF-A under hypoxic conditions, simulating the initiation of the angiogenic pathway, these molecules were also stimulated when these cells were stimulated with rWSP + VEGF-A, and, in addition, an increase in the production of VEGFR-1/sFlt1 (anti-angiogenic molecule) was observed. However, we observed no effect on sVEGFR-2 or mEndoglin (pro-angiogenic molecules). VEGF-A production has been reported in other models in relation to those produced by other nematodes such as *T. spiralis* or lymphatic filaria infections [25,26,33,34].

Likewise, VEGF-A production is also related to the activation of the fibrinolytic system [35], which is able to release VEGF pools, so, hypothetically, its stimulation could contribute to angiogenesis. VEGF-A production is also activated by rWSP [19], which activates plasminogen and generates plasmin, but when maintained for long periods of time leads to a pathogenic mechanism that damages the endothelium [36]. In addition, the increase in VEGF-A stimulates endothelial permeability and inflammation [37]. The anti-angiogenic pathway, which appears to be stimulated by rWSP, is supported by the proinflammatory effect also produced by *Wolbachia* sp. where an increase in iNOS expression and in the production of TxB_2_ and LTB_4_ has been reported [5,6,7,38].

In relation to the cellular processes of cell proliferation and migration analysed in this study, which are a consequence of the pro-angiogenic process [39,40], they are not stimulated by rWSP, which is consistent with the previous results of an anti-angiogenic effect. Furthermore, we have observed in our model a decrease in pseudo-capillary formation in the same way as previously evidenced [19]. However, other studies have shown that this process is also altered when the *Wolbachia* sp. load decreases in *D. immitis* adult worms treated with doxycycline compared to untreated worms [27], which could indicate the existence of other alternative pathways in the inflammatory process caused by the release of *Wolbachia* sp. into the bloodstream.

These results, with a clear anti-angiogenic effect, may also be related to the pro-inflammatory, pro-thrombotic, and anti-fibrinolytic processes produced by Wolbachia sp. through rWSP, as demonstrated in previous studies [2,5,6,7]. All would lead to the fact that *Wolbachia* sp., when released into the bloodstream of the canine host, does not promote *D. immitis* survival. In clinical practice, these results could be important because, by detecting the production of anti-angiogenic factors in the canine host with cardiorespiratory symptoms and with a positive antigen test for *D. immitis* (whether or not it has microfilariae in the blood), the presence of *Wolbachia* sp. in the bloodstream could be detected indirectly, showing that the disease is at an advanced stage, which is to be taken into account in the personalised treatment of the infected animal.

## 5. Conclusions

In summary, this study demonstrates that rWSP stimulates the expression of anti-angiogenic factors and not pro-angiogenic ones, as well as the ability not to form pseudo-capillaries in vitro, and does not stimulate cell migration and proliferation, all of which are linked to the pro-angiogenic effect. With these results, we can see that *Wolbachia* sp. is linked to the anti-angiogenic process, and that it is one more pathway to be taken into account together with the activation of other processes related to inflammation and fibrinolysis, which do not seem to favour the survival of *D. immitis* in the vascular stream.

## Data Availability

The raw data supporting the conclusions of this article will be made available by the authors on request.
